# Adaptive Spiral Tool Path Generation for Diamond Turning of Large Aperture Freeform Optics

**DOI:** 10.3390/ma12050810

**Published:** 2019-03-08

**Authors:** Dongfang Wang, Yongxin Sui, Huaijiang Yang, Duo Li

**Affiliations:** 1 Changchun Institute of Optics, Fine Mechanics and Physics, Chinese Academy of Sciences, Changchun 130033, China; suiyx@sklao.ac.cn (Y.S.); yanghj@sklao.ac.cn (H.Y.); 2 University of Chinese Academy of Sciences, Beijing 100049, China; 3 Changchun National Extreme Precision Optics Co., Ltd., Changchun 130033, China; 4 Centre for Precision Engineering, Harbin Institute of Technology, Harbin 150006, China; duo.kevin.li@gmail.com

**Keywords:** freeform optics, slow tool servo, tool path generation, large aperture optics

## Abstract

Slow tool servo (STS) diamond turning is a well-developed technique for freeform optics machining. Due to low machining efficiency, fluctuations in side-feeding motion and redundant control points for large aperture optics, this paper reports a novel adaptive tool path generation (ATPG) for STS diamond turning. In ATPG, the sampling intervals both in feeding and cutting direction are independently controlled according to interpolation error and cutting residual tolerance. A smooth curve is approximated to the side-feeding motion for reducing the fluctuations in feeding direction. Comparison of surface generation of typical freeform surfaces with ATPG and commercial software DiffSys is conducted both theoretically and experimentally. The result demonstrates that the ATPG can effectively reduce the volume of control points, decrease the vibration of side-feeding motion and improve machining efficiency while surface quality is well maintained for large aperture freeform optics.

## 1. Introduction

Most currently conventional optical devices from 2D optical design methods are usually rotationally symmetric. While systems with such optics perform well in numerous applications, the requirement of high energy efficiency or aberration correction often cannot be satisfied with the same optics because of its inherent symmetry limitations on geometry. In order to meet those kinds of demand, 3D optical design methods have been developed, such as the simultaneous multiple surface (SMS) 3D method [1], supporting the quadric method [2]. In general, the results of a 3D design method are nonrotational, nonlinear asymmetric surfaces, known as freeform surfaces. With the development of 3D optical design methods, large aperture freeform optics are increasingly used in both non-imaging and imaging optical systems due to its capacity of improving optical performance [3,4,5,6]. There are three ultra-precision machining processes, namely fast tool servo (FTS), slow tool servo (STS) and diamond milling frequently used to produce optical freeform surfaces. STS machining has the advantages of fast setting-up and a large degree of freedom in processing, especially for freeform surfaces with large deviation [7,8]. However, the tool path generation and tool shape compensation must be conducted carefully for efficiently producing contoured surface with submicron form accuracy and nano-scale roughness or less.

Over the past decades, much research work has been conducted on tool path generation for diamond turning of freeform optics, including angle sampling strategy, spiral path and tool radius compensation. Fang et al. [9] used non-uniform rational basis spline (NURBS) surface to represent a freeform surface, and carried out the compensation and optimized values for tool geometry. Gong et al. [10] calculated the non-zero rake angle tool position directly from symbolic computation. In addition, the authors [11] projected space Archimedean spiral onto freeform surface along the normal direction of the base surface to get the diamond tool path. Wang et al. [12] machined a toric surface by generating a spiral tool path with the constant-angle method, and analyzed the different entrance parameters algorithm on Position–Velocity–Time (PVT) interpolation. Zhu et al. [13] proposed the adaptive tool servo (ATS) mode that the sampling angle and feedrate were actively adjusted at any cutting point to adapt shape variation of the desired surface.

During the conventional multi-axis computerized numerical control (CNC) milling of freeform surfaces, it is crucial to adapt the side-feeding motion and forward sampling of tool motions to surface shape variations to achieve uniform surface quality with maximum efficiency [13]. Koren et al. [14] used a non-constant offset of the previous tool path to guarantee the cutter moving in an un-machined area and without redundant machining. However, this method is not suitable for STS because the non-constant offset of the spiral path will lead to additional motion of the lateral feeding axis (*x*-axis) when it follows the *c*-axis. Due to the limitation of the dynamic response of the servo axis, this will lead to the following error and vibration.

Generally, the following problems exist in tool path generation algorithm for slow tool servo diamond turning of large aperture freeform optics:

Current angle sampling strategies are still constant-angle and constant arc-length methods [15,16], which are aimed at path generation of axisymmetric surfaces. However, the curvature of a freeform surface is not uniform on the whole part and the strategy of constant-angle or constant arc-length cannot adapt to the change of freeform surface shape. Therefore, the uniform sampling will inevitably lead to an uneven distribution of surface accuracy [11,17]. Especially for large aperture optical surfaces, when the constant-angle method is applied, the surface quality of central region is better than that of the outer region due to the fact that the point density of outer region is relatively sparser than those on the central region. Therefore, in order to ensure the allowable interpolation error on the outer region, a large number of sampling points are needed on the outer region of the surface, which results in redundant control points on the center region. Moreover, the volume of points generated by this method probably exceeds the storage capacity of CNC system.

The cutting residual is caused by the tool radius envelope along the feeding direction, which is regarded as surface roughness. Kwok et al. [18] and Yu et al. [19] developed the model for predicting the residual error in STS, indicating that the residual error is not only dominated by the tool nose radius and feedrate, but also highly dependent on local surface curvature. Currently, the commonly adopted constant feed in STS leads to a heterogeneous distribution of the cutting residual error, especially for freeform optics with large curvature variation. To guarantee the cutting residual within a tolerance level, machining of the entire whole surface is required to maintain the minimum feedrate, which is not necessary for other regions and significantly reduces the machining efficiency.

Current tool path generation methods do not consider the dynamic performance of machine tools. In traditional machining of rotational symmetrical surfaces, the feed axis and spindle are independent, so the dynamic performance of machine tool is not considered. However, when a freeform surface is machined by STS diamond turning, the feed axis and the spindle should maintain a strict spatial position relationship. Therefore, an optimal tool path is very important. Although the ATS method proposed by Zhu [13] can produce control points with a uniform distribution of surface profile errors, and much CAM software used in milling which could be ported to turning can output a constant load or constant scallop height tool path [20,21]. However, because they produce irregular spiral paths, this will bring additional fluctuations to the feed axis, reducing the machining surface quality.

In this paper, a novel path generation of STS diamond turning for large aperture freeform optics; namely, adaptive tool path generation (ATPG) is proposed. Firstly, the principle of the novel generation method is introduced and the path generation algorithm is presented in detail with analysis of the motion characteristics of machine tools. The results show that, on the premise of satisfying the machining accuracy, the proposed algorithm can reduce the volume of control points, and improve the dynamic characteristics of machine tools and machining efficiency. Finally, both theoretical analysis and experimental comparison of the algorithm are conducted.

## 2. Basic Principle of Adaptive Tool Path Generation

**Figure 1:**
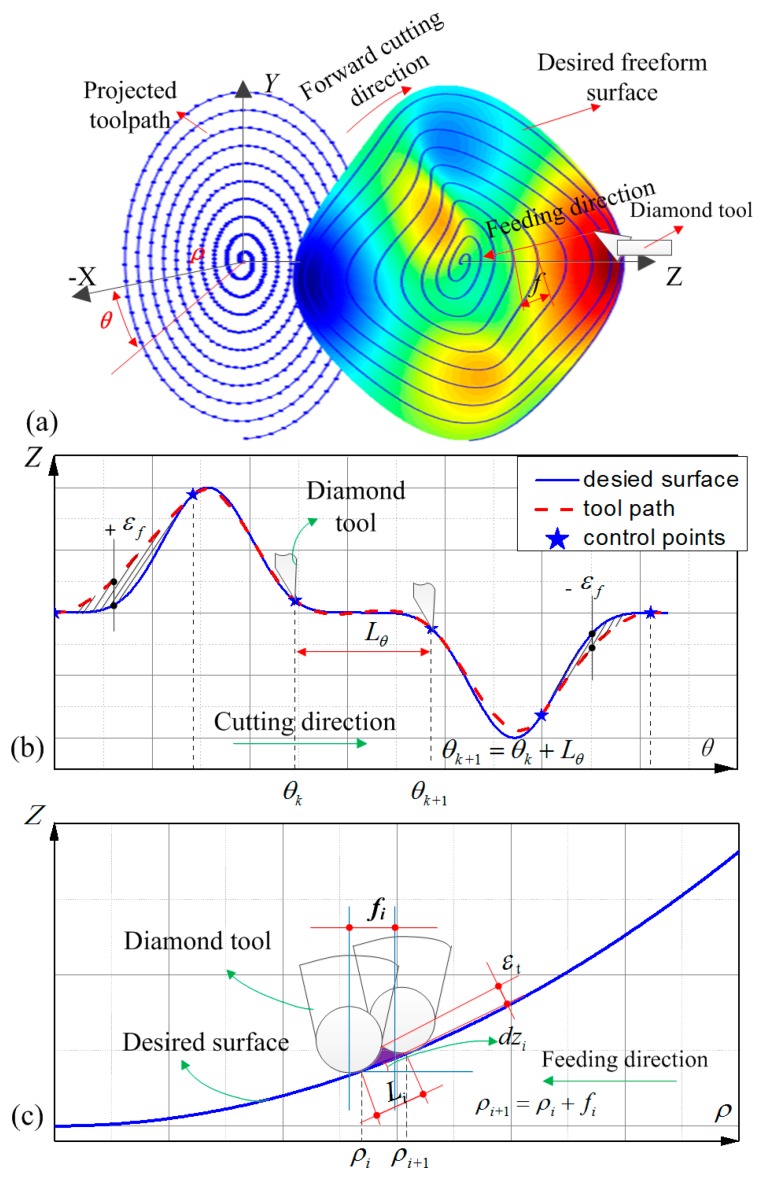
Schematic of principle of slow tool servo (STS), (**a**) the relative motions; (**b**) error in forward cutting direction and (**c**) error in feeding direction.

The principle of diamond tool turning freeform optics is that the diamond tool contours the complex surface along the spiral path as shown in Figure 1a. The stroke of the diamond tool is synchronized to the angle and radial position of freeform surface on the machine’s spindle. The forward and reverse motion is achieved by *z*-axes of machine tools (STS).

There are two intrinsic errors in STS machining, namely interpolation error and cutting residual as shown in Figure 1b,c [22]. In the forward cutting direction, the deviation between the desired surface and the tool trajectory leads to the interpolation error and the error is highly dependent on the distance *L*_θ_ between the two consecutive control points as well as the local surface profile. Generally, most diamond machine tools offer PVT spline interpolation for STS, which creates a path known as a cubic Hermite Spline. Therefore, for a given interpolation tolerance ε*_f_*, the algorithm adaptively adjusts the angle interval *L*_θ_ according to the error of Hermite interpolation to ensure that the error is within the range [−ε*_f_*_,_ + ε*_f_*], so that the error is homogeneously distributed in the whole cutting direction.

**Figure 2:**
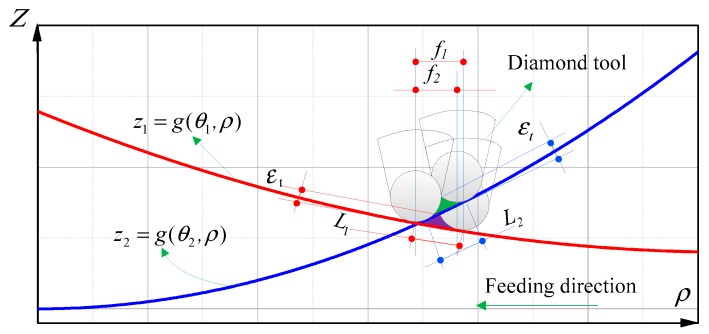
Schematic of feed variation with angle on the same radius for freeform surface.

**Figure 3:**
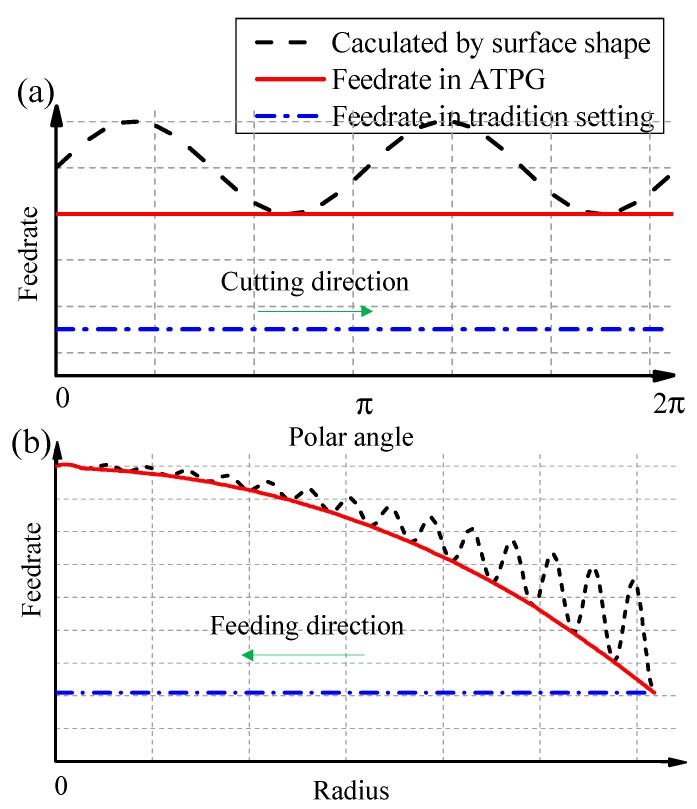
Schematic of feedrate in different methods. (**a**) Feedrate in one revolution of spiral path; (**b**) Feedrate along the radius on the whole surface.

In the feeding direction, in order to improve the machining efficiency, the feedrate should be adjusted with surface curvature. Because the curvature of a freeform surface not only changes with radius, but also changes with polar angles, as shown in Figure 2. The curvature of a freeform surface at the polar angle θ_1_ and θ_2_ of the same radius is different, so the calculated feedrate *f*_1_ and *f*_2_ on the same radius are different, which leads to the position of diamond tool changing with spindle angle in the process of feeding. As a result, micro-fluctuations are generated in the process of machining, which would deteriorate the surface quality of machining, just as shown in Figure 3a. The dash line denotes the feedrate calculated by the surface shape (In fact, it is the feedrate in ATS). In contrast, ATPG sets the feedrate to a constant equal to the minimum value of this revolution, which can restrain the fluctuations of feedrate with angles. Therefore, the final feedrate only changes with radial position. Furthermore, if the feedrate varies dramatically along the radius, the feedrate will be re-fitted with a smooth curve to suppress the vibration in the feeding direction, just as shown by the red solid line in Figure 3b. It should be noted that the blue dash dot line donates the feedrate in the traditional machining mode, which maintains the minimum feedrate to guarantee the cutting residual on the whole surface.

Although the feedrate in ATPG is slightly smaller than ATS, it is still larger than the traditional settings. The advantages of this setting are that it not only improves the machining efficiency, but also suppresses the micro-fluctuation caused by surface shape. Although this will result in a slight heterogeneous distribution of interpolation error at different angles on the same radius, it is almost negligible, and the advantage is to increase the stability of the machine tool.

## 3. Tool Path Generation for STS Diamond Turning

In the traditional STS diamond turning, the machined surfaces have to be evaluated after the machining process which gives a high risk of the machined surface failing to meet the contour accuracy. The surface generation algorithm is not only the generation of a diamond tool path, but also a predictive method to analyze the contour accuracy by using different cutting strategies.

**Figure 4:**
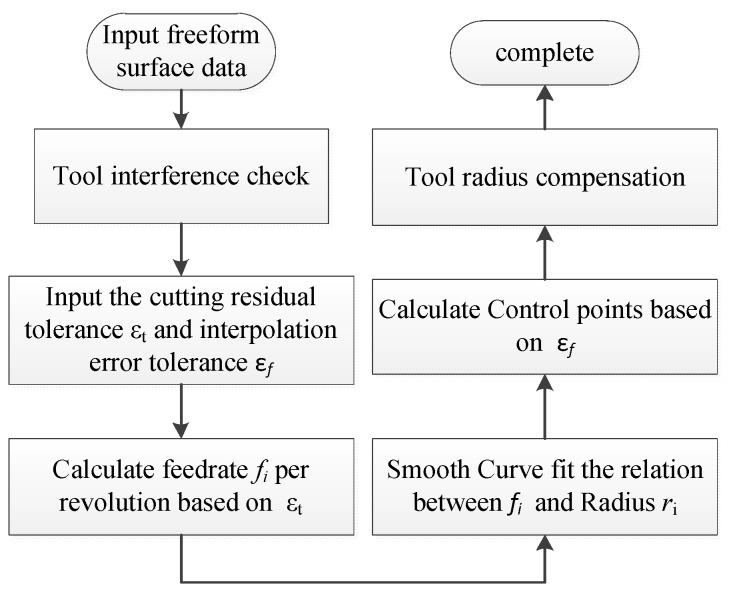
The general process of ATPG for STS.

Figure 4 shows the general process of ATPG, including five steps to generate the final control points: (1) Tool interference check; (2) Determination of the feedrate for every revolution of the spiral path; (3) Determination of smooth curve fitting the relationship between feedrate and radius; (4) Determination of control points in the cutting direction with the constraint of interpolation error; (5) Tool radius compensation.

### 3.1. Tool Interference Check

Due to the geometrical complexity of freeform surfaces, it is necessary to check tool interference, and the sectional curve method [9] is used to check the tool parameters before machining, which decomposes the whole surface into two-dimensional sectional curves and integrated the tool parameters from curves.

### 3.2. Determination of the Feedrate for Every Revolution of the Spiral Path

The coordinate system of the workpiece for STS machining is shown in Figure 1a. The desired surface can be expressed as *z* = *f*(*x*,*y*), which can be also expressed as *z* = *g*(θ,ρ) by coordinate transformation.

Let’s suppose that the radius corresponding to the *N_i_*-th revolution of the spiral path is ρ*_i_* at the angle θ*_i_* = 2π*i*, so the corresponding curvature at this revolution can be defined as [23]:

(1) $$K_{i}=\min\left\{{\frac{\partial^{2}g/\partial \rho^{2}}{{\left[1+\partial g/\partial \rho \right]}^{\frac{3}{2}}}|}_{\rho =\rho_{i}},\forall \theta \in \left[0^{{}^{\circ}}{,360}^{{}^{\circ}}\right)\right\}$$

where *K_i_* means the minimum curvature of the cross-sectional profile passing through the rotation center over the whole revolution.

By approximating the local profile as a segment of arc, the distance *L_i_* (Figure 1c) between the two consecutive revolutions *N_i_* and *N_i_*_+1_ on the desired surface with respect to a given cutting residual error tolerance ε_t_ (Figure 1c) can be estimated by [14]:

(2) $$L_{i}=\sqrt{\frac{8R_{t}\varepsilon_{t}}{1-R_{t}K_{i}}}$$

where *R*_t_ denotes the nose radius of a diamond tool.

At the same time, we define the slope of the cross-sectional profile *dz_i_* (Figure 1c) at the radius ρ*_i_* as:

(3) $$dz_{i}=\min\left\{{\frac{\partial g}{\partial \rho }|}_{{\rho =\rho }_{i}},\forall \theta \in \left[0^{{}^{\circ}}{,360}^{{}^{\circ}}\right)\right\}$$

As shown in Figure 1c, the feedrate *f_i_* for the *N_i_*-th revolution in the projected tool path and radius ρ*_i_*_+1_ corresponding to the *N_i_*_+1_-th revolution in the projected tool path can be approximately expressed as:

(4) $$f_{i}=\frac{L_{i}}{\sqrt{1+dz_{i}{}^{2}}}$$

(5) $$\rho_{i+1}=\rho_{i}+f_{i}$$

The first revolution can be set as:

(6) $$N_{1}=0,\rho_{1}=d/2$$

where *d* is the inner diameter of the desired surface, and if the desired surface is started from the center, *d* = 0.

Since there is only one parameter ρ*_i_*_+1_ unknown, it can be calculated by numerical iteration solving Equations (1), (2) and (6). Thus, we can sequentially obtain series of number pairs (*N_i_*,ρ*_i_*).

### 3.3. Determination of Smooth Curve about the Relationship between Feedrate and Radius

Obviously, the curve of series of number pairs (*N_i_*,ρ*_i_*) is related to the characteristics of side-feeding motion of machine tool, especially for the machine tool whose spindle is mounted on the *x*-axis. The drastic fluctuations in the curve may cause the side-feeding acceleration of the machine tool to exceed the allowable range, which may result in the failure of the machining process. Therefore, some drastic fluctuations of the number pairs (*N_i_*,ρ*_i_*) should be limited, if necessary. Hence, we use a continuous and smooth curve to approximate the curve of number pairs (*N_i_*,ρ*_i_*) and the feedrate obtained by fitting should not be greater than number pairs (*N_i_*,ρ*_i_*), just as shown by the red solid line in Figure 3b. After that, we can obtain the functional relationship between the radius ρ and revolution number *N*, which can be expressed as:

(7) ρ=ℓ(N)

where *N* denotes revolution of spiral, and it is not necessary to be an integer.

### 3.4. Determination of the Control Points in Cutting Direction

Let us assume that the *k*-th cutting point *P_k_*(θ*_k_*,ρ*_k_*,*z_k_*) in the *N_k_*-th revolution of the spiral path, so the corresponding angle θ*_k_* can be expressed by

(8) $$\theta_{k}=2\pi \left(N_{k}-\left[N_{k}\right]\right)$$

where [∙] denotes rounding operation.

Because the spiral radius increment and angle increment are very small between the two consecutive control points in the actual tool path, an arc can approximate the segment spiral path. Hence, the tool spiral path that passes through the point *P_k_*(θ*_k_*,ρ*_k_*,*z_k_*) can be approximated by the curve *z* = *g*(θ,ρ*_k_*), and the parameter ρ*_k_* is already known, so the curve is a function of one variable, which can be expressed approximately as:

z = ϕ(θ) = g(θ, ρ*_k_*), θ ∈ (θ*_k_* − δ,θ*_k_* + δ) (9)

where δ is a small number, and ϕ(∙) denotes the operator notation of one variable function.

PVT mode provides an excellent contouring capability because it takes the interpolated commanded path exactly through the control points. It generates a path known as a Hermite spline. Hence, according to the error of piecewise cubic Hermite interpolation, the relationship between the interpolation error tolerance ε*_f_* and angle increment *L*_θ_ between the two consecutive control points *P_k_*(θ*_k_*,ρ*_k_*,*z_k_*) and *P_k_*_+1_(θ*_k_*_+1_,ρ*_k_*_+1_,*z_k_*_+1_) can be constructed by [24]:

(10) $$\varepsilon_{f}\leq \frac{L_{\theta }^{4}}{384}\underset{\theta_{k}\leq \theta \leq \theta_{k+1}}{\max}|\phi^{\left(4\right)}\left(\theta \right)|$$

where the curve *z* = ϕ(θ) is supposed ϕ(θ) ∈ *C*^4^[θ*_k_* − δ, θ*_k_* + δ], and ϕ^(4)^(∙) denotes the 4th derivative.

The following relation is also established as:

(11) $$\theta_{k+1}{=\theta }_{k}+L_{\theta }$$

so the unknown parameters *L*_θ_ and θ*_k_*_+1_ be derived from Equations (10) and (11) by numerical calculation.

The corresponding revolution *N_k_*_+1_ for *P_k_*_+1_ can be obtained by:

(12) $$N_{k+1}=N_{k}+L_{\theta }/2\pi$$

The other parameters ρ for *P_k_*_+1_ can be obtained by:

(13) $$\rho_{k+1}=\ell (N_{k+1})$$

(14) $$z_{k+1}=g{(\theta }_{k+1},\rho_{k+1})$$

Thus, the coordinate of point *P_k_*_+1_(θ*_k_*_+1_,ρ*_k_*_+1_,*z_k_*_+1_) can be obtained by combining the equations from Equation (8) to Equation (14). By conducting the iterative steps with respect to *N*_1_ = 0, the whole toolpath can be adaptively resampled.

### 3.5. Tool Radius Compensation

**Figure 5:**
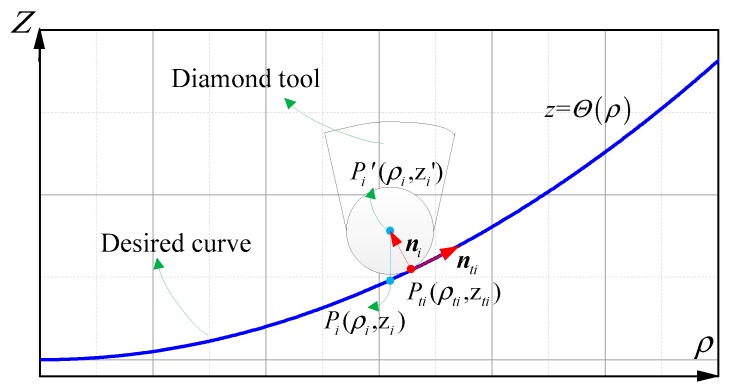
Schematic of tool radius compensation in the *z*-direction.

Traditionally, there are two methods of tool nose radius compensation for diamond turning, *xz*-direction compensation [9,25] and *z*-direction compensation [10]. Generally, the *xz*-direction tool compensation is accomplished by the normal vector of the machined surface and cutting plane; however, the direction of normal vector of the freeform in the cutting plane periodically changes with angle, resulting in a slight periodic displacement of the tool in side-feeding motion. Especially for the machine tool whose spindle is mounted on the *x*-axis, the stability of side-feeding motion is particularly important to surface quality. Thus, we adopt the *z*-direction compensation in ATPG as shown in Figure 5.

Without loss of generality, *P_i_*(θ*_i_*,ρ*_i_*,*z_i_*) is the control point, and *P_i_′*(θ*_i_*,ρ*_i_*,*z_i_′*) is the corresponding coordinate of tool edge center, where *z_i_′* is the only unknown parameter. The curve that is a cross-sectional profile passing through the rotation center *O* and *P_i_* can be expressed as:

(15) $$z={g(\theta ,\rho )|}_{\theta =\theta_{i}}=\Theta \left(\rho \right)$$

*P*_t*i*_(θ*_i_*,ρ_t*i*_,*z*_t*i*_) is assumed as the corresponding foot point on the curve *z* = Θ(ρ) of the *P_i_′*(θ*_i_*,ρ*_i_*,*z_i_′*), where ρ_t*i*_*, z*_t*i*_ are the unknown parameters as shown in Figure 5. According to the geometric constraint, assuming that a round edged diamond tool with a zero rake angle is adopted, the following relations can be obtained:

(16) $$\left\{ \begin{array}{l} \left\|{\bar{P_{i}{}^{\prime }P}}_{ti}\right\|=R_{t}\\ n_{i}\cdot n_{ti}=0\\ z_{ti}=g{(\theta }_{i},\rho_{ti}) \end{array}\right.$$

where‖∙‖ denotes the Euler distance between any two points, and ***n****_i_* is the normal vector of the curve *z* = Θ(ρ) at the point *P*_t*i*_(θ*_i_*,ρ_t*i*_,*z*_t*i*_), which can be expressed as:

(17) $$n_{i}=\left(\rho_{i}-\rho_{ti},z_{i}{}^{\prime }-z_{ti}\right)$$

where ***n***_t*i*_ is the tangent vector of the curve *z* = Θ(ρ) at the point *P*_t*i*_(θ*_i_*,ρ_t*i*_,*z*_t*i*_), which can be expressed as:

(18) $$n_{ti}={(1,\frac{\partial \Theta }{\partial \rho })|}_{\rho =\rho_{ti}}$$

Thus, according to Equations (15), (17) and (18), Equation (16) can be solved by Newton’s iteration method.

After the tool radius compensation, the whole tool path control points are generated.

## 4. Theoretical Investigation

### 4.1. Demonstration of Surface Generation in ATPG

**Figure 6:**
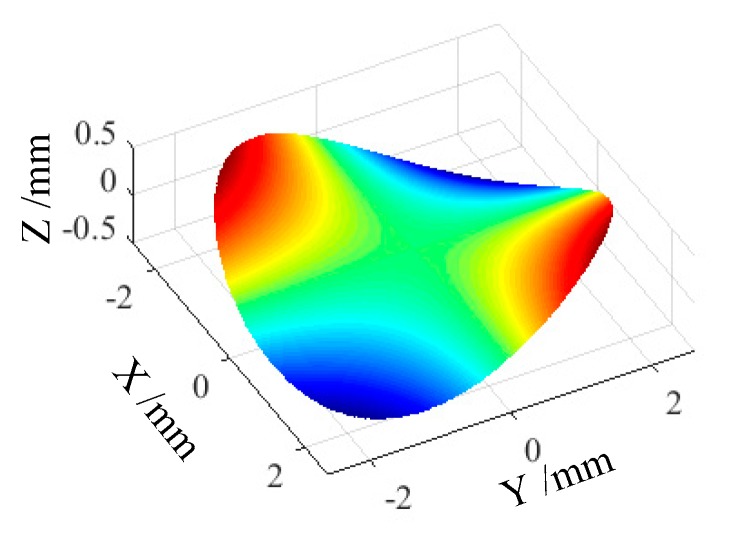
Schematic of desired surface (astigmatic surface).

To demonstrate the process of this novel method, a typical astigmatic surface was employed for investigation as shown in Figure 6, which can be mathematically described as *z* = *K*ρ^2^sin(2θ). To clearly show the difference between the ADPT and conventional method, the aperture of the workpiece was set as 5 mm. and the amplitude was set as 1mm. The tool nose radius was set as *R_t_* = 1.0 mm with a zero degree rake angle, and the tool parameters have been checked by tool interference check.

Tolerances of interpolation error and cutting residual were set as ε*_f_* = 100 nm and ε*_t_* = 100 nm, respectively, based on the optical requirements.

**Figure 7:**
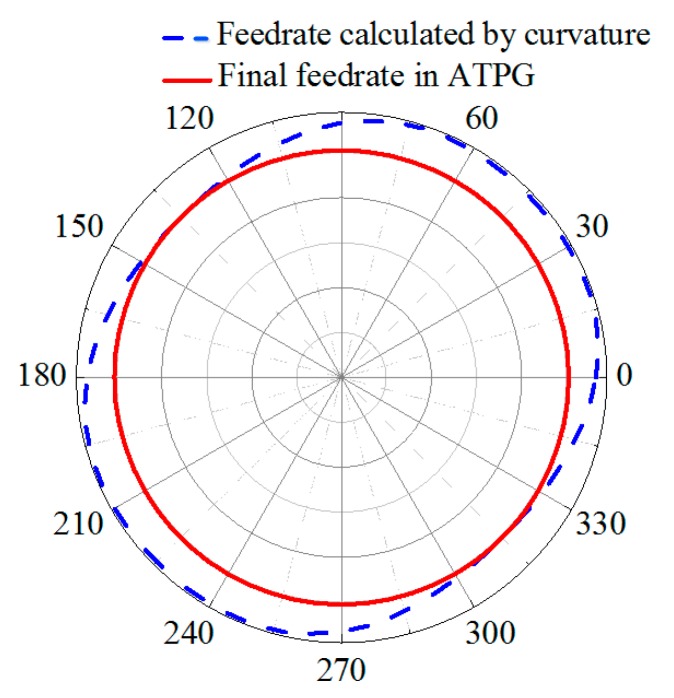
Schematic of feed calculation process at ρ = 2 mm.

We firstly calculated the feedrate for each revolution, and Figure 7 showed the calculation process of feedrate for radius ρ = 2 mm (reference radius). Firstly, according to the curvature of the cross-sectional profile passing through the rotation center and the equations mentioned above, the feedrate at different angles was calculated and shown in the blue dash line. It can be seen that the feedrate is different at different angles. This means that the motion of the *x*-axis was different with the position of a *c*-axis at this radius, which would cause micro-fluctuations of *x*-axis. In order to suppress these fluctuations and guarantee the accuracy of cutting residual, we set the feedrate equal to the minimum feedrate, as shown in the red line, ensuring that the *x*-axis remained uniform motion in all positions of *c*-axis on this radius. As in so many other revolutions, we can get *f_i_* and *N_i_* along the feeding direction on the whole surface.

**Figure 8:**
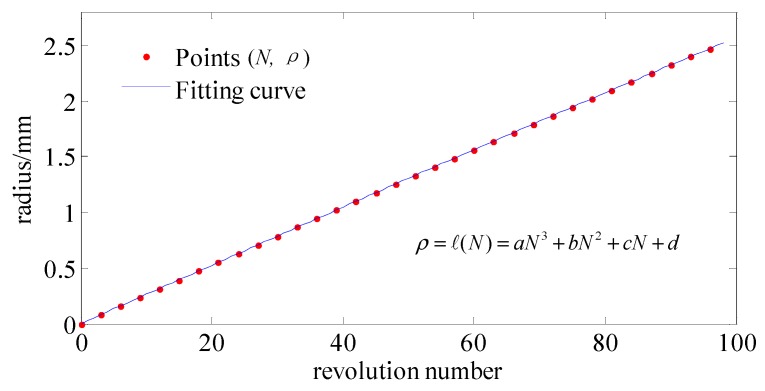
Curve fitting for (*N_i_*,ρ*_i_*).

After obtaining series of number pairs (*N_i_*,ρ*_i_*), which are shown in Figure 8 with the red dotted line, in order to suppress drastic fluctuations in the side-feeding motion of machine tool, we used a continuous and smooth curve to approximate these number pairs (*N_i_*,ρ*_i_*) by a numerical curve fitting method. This curve model can be any mathematical function. For this case, after comparing the fitting residuals of various functions, cubic polynomials were suitable because its residuals are the smallest, and it can be expressed by:

(19) $$\rho =\ell (N)=aN^{3}+bN^{2}+cN+d$$

where polynomial coefficient *a* = −4.9469 × 10^−8^, *b* = −8.774 × 10^−7^, *c* = 0.0263, and *d* = −1.141 × 10^−4^. The sum of squares error (SSE) is 2.082 × 10^−7^; this means that goodness of fit is very high.

Thus, we can obtain the whole surface control point coordinates by combining the equations from Equations (8) to (14).

After the tool radius compensation with solving Equation (16), the whole tool path control points were finished.

### 4.2. Characterization of Motion in ATPG

**Figure 9:**
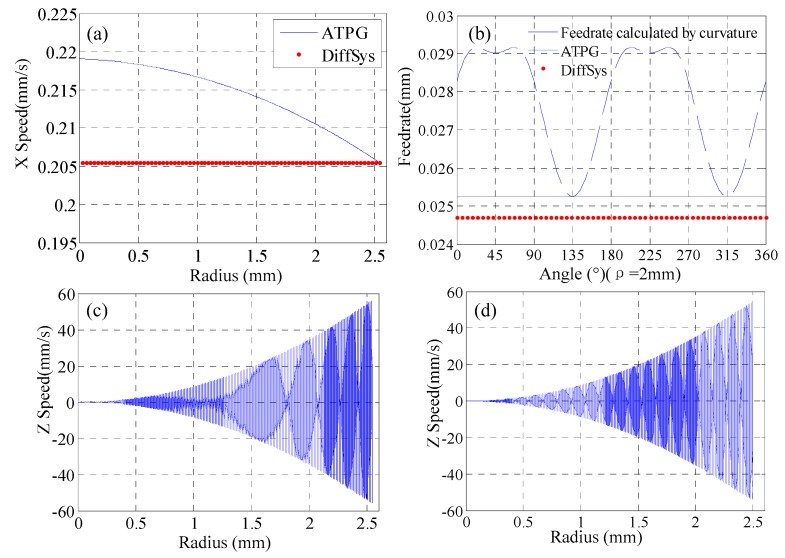
Features of the motion (**a**) side-feeding motion in the feeding direction; (**b**) feedrate with angles at ρ = 2mm in the cutting direction, translational motion along the *z*-axis in (**c**) ATPG and (**d**) DiffSys.

To characterize the motions of the machine tool, the sampling intervals for the *x*-axis and *z*-axis were extracted. The spindle speed was set as 500 rpm. The velocity for each axis is as shown in Figure 9. It is noted that the sampling strategy in the region near the center of rotation follows the constant angle strategy due to the too large interval for the given interpolation error.

To have a comparison, the tool path was generated by both ATPG and conventional method, which is calculated by DiffSys (a commercial and professional CAM software for ultra-precision turning, version 3.98, WESTERN ISLE, North Wales, GB). DiffSys follows the constant-angle strategy which was based on the interpolation tolerance and the minimum radius of curvature of the part. It is clear that in the feeding direction the *x*-axis speed varies smoothly according to surface shape in ATPG from Figure 9a, and the speed does not present micro fluctuations during the whole process. This is due to the smooth curve fitting and no compensation on the *x*-axis direction in ATPG. Because the feedrate in DiffSys is defined by the minimum radius of curvature of the part, the feedrate of ATPG is greater than that of DiffSys on the whole part. Meanwhile, although in order to suppress the micro fluctuations of motion in the cutting direction, ATPG takes the minimum feedrate calculated according to the surface shape in every spiral path, it is still higher than that of DiffSys. Through Figure 9a,b, it is clear that the machining efficiency is improved in ATPG. Figure 9c,d show the speeds of the two methods in the *z*-axis, and there is no obvious difference in the *z*-direction.

**Figure 10:**
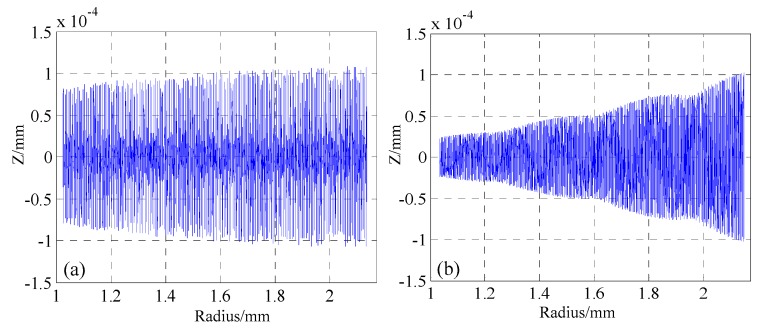
Characteristics of the generated surface error maps along the toolpath in (**a**) ATPG and (**b**) DiffSys.

We computed the intermediate “way-points” for each axis for each point along the spline path by Hermite spline interpolation. After subtracting the desired surface, error maps of the theoretically generated surface for ATPG and DiffSys are illustrated in Figure 10. It can be seen that all interpolation errors are restricted in [−ε*_f_*_,_ +ε*_f_*], and the interpolation errors are homogeneously distributed over the entire surface. For DiffSys which adopts the constant-angle methods, the surface quality of central region is better than that of the outer region, and in order to ensure the allowable interpolation error on the outer region, a large number of sampling points are needed on the outer region of the surface, which results in redundant control points on the center region. In this case, the number of control points in ATPG (3,920) was only about 64% of that in DiffSys (6061). Therefore, the machining time in DiffSys would be much longer than that in ATPG to have similar machining accuracy.

## 5. Experimental Results and Discussion

### 5.1. Experimental Setup

The astigmatic (AST) surface was machined for investigating the performances. Considering the testing ability of the interferometer, the aperture of the workpiece was 50 mm, the amplitude was set as 4.8 μm, respectively. To have a comparison, the tool path was generated by both ATPG and DiffSys, and DiffSys follows the constant-angle strategy that was based on the interpolation tolerance and the minimum radius of curvature of the part. The feedrate in DiffSys was set as *f* = 8.9 μm/rev, which was based on the surface finish specification and the maximum slope of the part, and the tool radius compensation in DiffSys was set along the *z*-direction as well.

In order to validate the performance of ATPG for large aperture optical surfaces, an off-axis paraboloid (OAP) with an aperture of 213 mm was also machined, where the vertex curvature radius R was 915 mm and off-axis magnitude was 915 mm. We compared the programs generated both by ATPG and Diffsys for the same setting rule.

**Figure 11:**
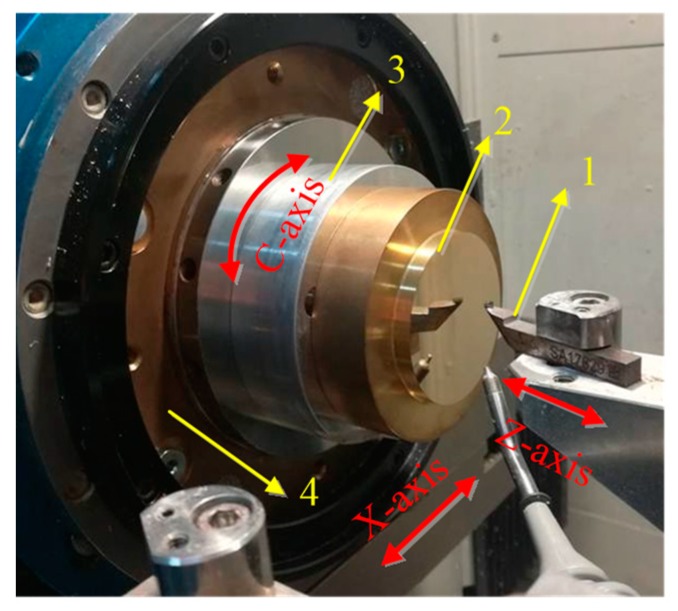
Hardware configurations of AST surface machining, where (1) diamond tool; (2) workpiece; (3) fixture; (4) spindle.

**Figure 12:**
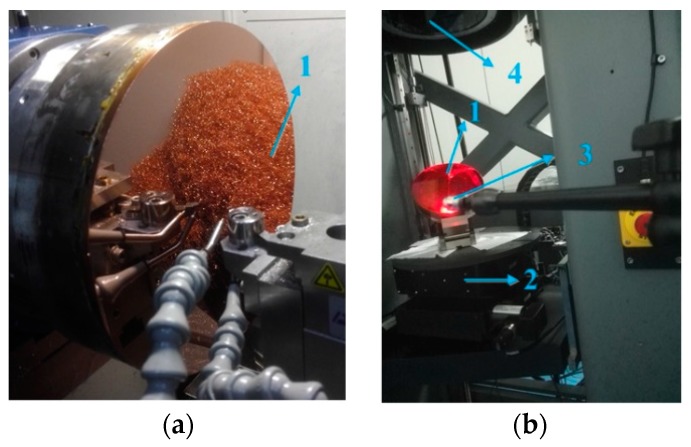
(**a**) Hardware configurations of off-axis paraboloid (OAP) machining and (**b**) form test, where (1) workpiece; (2) adjustment mechanism; (3) standard ball; (4) planar interferometer.

The cutting experiments were performed on a CNC ultra-precision lathe (Precitech Nanoform^®^ 250 ultra, Keene, NH, USA). The configurations of this experiment were shown in Figure 11 and Figure 12a. A natural single crystal diamond tool with a nose radius of 1.04 mm and a zero rake angle (Contour Fine Tooling, Hertfordshire, UK) was used in this experiment. Before machining, the diamond tool parameters have been checked according to the sectional curve method. The workpiece materials are brass and oxygen-free high-conductivity copper (OFHC), respectively.

After turning, a laser Interferometer (Zygo, MST, Middlefield, CT, USA) was employed to measure the machined surface form error. The AST surface was tested directly by the interferometer and the OAP was tested though optical properties of quadric surfaces as shown in Figure 12b. A white light interferometer (Bruker, ContourGT-K1, Madison, WI, USA) was employed to measure surface roughness.

### 5.2. Results and Discussion

**Figure 13:**
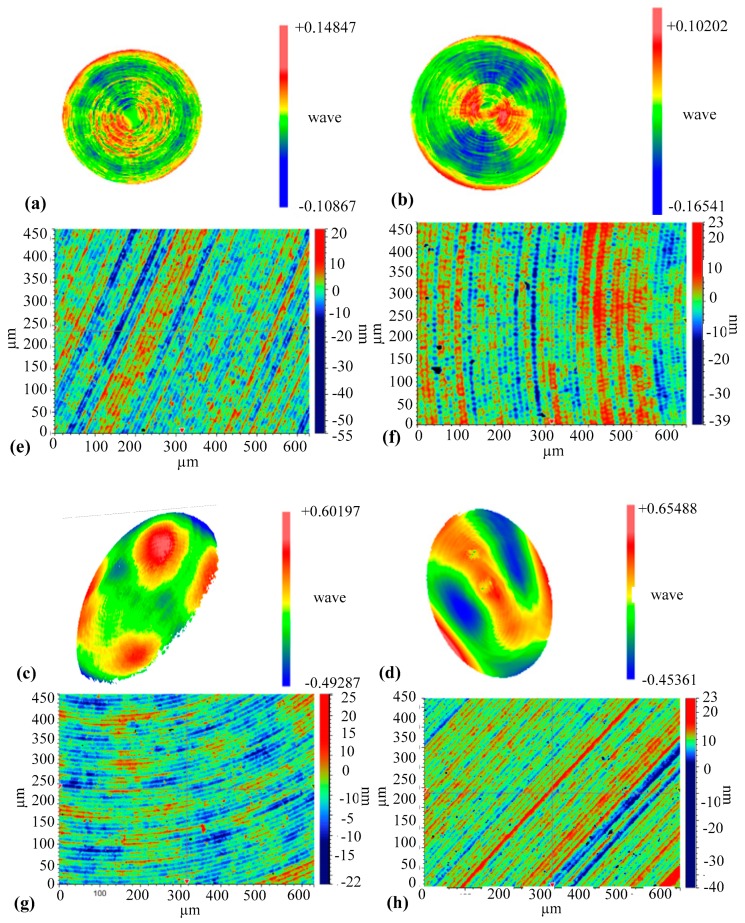
The form error obtained by (**a**) ATPG (AST), (**b**) DiffSys (AST), (**c**) DiffSys (OAP), (**d**) ATPG (OAP); and the roughness of the turned surfaces obtained by (**e**) ATPG (AST); (**f**) DiffSys (AST); (**g**) DiffSys (OAP); (**h**) ATPG (OAP).

After subtracting the design surface, the form errors for the two machined surfaces (AST) are respectively shown in Figure 13a,b. The roughness was measured by a white light interferometer and shown in Figure 13e,f, respectively. More details of the experimental results are summarized in Table 1. The total volume of control points for ATPG was 23,581, while that for DiffSys was 35,053. As shown in Table 1, it can be seen that the surface machined by ATPG is slightly better, but considering the fabrication and metrology uncertainty, there is no significant difference between the two methods for surface quality. Nevertheless, the number of control points generated by ATPG is 32.7% less than by DiffSys in this case. The machining efficiency is also improved, which is particularly important for large-aperture optical surfaces.

The machined OAP surfaces were measured through optical properties of quadric surfaces as shown in Figure 12b and further shown in Figure 13c,d; the roughness was shown in Figure 13g,h. Comparing the data in Table 1, the number of control points generated by ATPG is 52.5% less than by DiffSys in this case, and the roughness is still keeping the same level; this is very significant for large aperture optics to reduce the volume of NC program. Although the form errors exceed the preset value, it is mainly caused by low frequency components from the test data, which should be caused by clamping deformation. This is not the subject of this paper.

Generally speaking, more control points mean higher interpolation accuracy. However, as the number of points increases, the number of points is no longer the bottleneck for machining accuracy. Furthermore, because the new tool path makes the axis speed variation very small, the machine tool can adapt to a higher spindle speed. Hence, the comparison verifies the high machining efficiency of the ATPG for achieving the same surface quality. It is noted that the PV value of the form error and roughness is higher than the preset theoretical one. The reason is that, besides the accuracy of interpolation, factors that determine the surface form and roughness still include machine tool vibration, tool wear, lubrication and clamping deformation, etc.

## 6. Conclusions

This research proposes a novel adaptive tool path generation (ATPG) for freeform optics to improve machining efficiency. The main conclusions are as follows:

- With consideration of machine tool motion characteristics, an adaptive tool path generation is investigated, including establishing the functional relationship between spiral radius and revolution number with respect to cutting residual, smooth curve fitting and solving cutter contact points in the cutting direction with the consideration of interpolation error and tool radius compensation.
- A theoretical investigation for ATPG is conducted. The results indicate that the smooth curve produced by the spiral radius and revolution number can effectively suppress vibration of side-feeding motion, which can make the machining operated at a higher spindle speed.
- A comparison investigation on surface generation in ATPG and DiffSys was experimentally conducted on typical freeform surfaces. By adopting ATPG, the volume of tool path control points and machining time are effectively reduced, while surface form error as well as roughness is maintained. Therefore, the proposed ATPG significantly increases the machining efficiency for large aperture freeform optics.

## Figures and Tables

**Table 1:** Result of the experiments.

Item	AST		OAP	
	ATPG	DiffSys	ATPG	DiffSys
Number of control points	23,581	35,053	281,141	592,541
Cutting time	5 min 30 s	8 min	1 h	2.2 h
PV error (nm)	163	169	701	693
Roughness *R*_a_ (nm)	4.8	4.9	4.6	4.5
